# Impact of weight loss and disease progression on survival in ALS: insights from a multidisciplinary care center

**DOI:** 10.1055/s-0045-1812029

**Published:** 2025-10-15

**Authors:** Mário Emílio Teixeira Dourado Junior, Laura Carvalheira Dourado, Glauciane Costa Santana, Sancha Helena de Lima Vale, Lucia Leite-Lais

**Affiliations:** 1 Universidade Federal do Rio Grande do Norte, Centro de Ciências da Saúde, Departamento de Medicina Integrada, Natal RN, Brazil.; 2 Universidade Federal do Rio Grande do Norte, Centro de Ciências da Saúde, Programa de Pós-Graduação em Nutrição, Natal RN, Brazil.; 3 Universidade Federal do Rio Grande do Norte, Hospital Universitário Onofre Lopes, Natal RN, Brazil.; 4 Universidade Federal do Rio Grande do Norte, Centro de Ciências da Saúde, Departamento de Nutrição, Natal RN, Brazil.

**Keywords:** Amyotrophic Lateral Sclerosis, Weight Loss, Disease Progression, Nutritional Status, Survival Analysis

## Abstract

**Background:**

Amyotrophic lateral sclerosis (ALS) is a multifaceted neurodegenerative disorder with a poor prognosis. Weight loss and malnutrition emerge as significant clinical features during disease progression.

**Objective:**

To explore how demographic and clinical characteristics relate to survival in ALS patients, emphasizing the role of weight loss percentage at the time of diagnosis.

**Methods:**

We conducted a retrospective study that used the database of a multidisciplinary ALS care center in the city of Natal, Brazil.

**Results:**

A total of 132 patients were included in the study. The mean age of the participants at symptom onset was of 56.9 years, and most of them were male (59.8%). Older age, bulbar onset, and faster disease progression were associated with weight loss ≥ 10% at diagnosis. Among 132 patients, 72% experienced death or tracheostomy, with a median survival of 34 months. Survival was notably reduced in patients aged ≥ 60 years, those with significant weight loss, rapid disease progression, or those submitted to gastrostomy. Weight loss and the rate of disease progression were the strongest predictors of reduced survival. Potential factors relating gastrostomy with reduced survival are discussed.

**Conclusion:**

The present study highlights the critical impact of weight loss and disease progression on survival in ALS patients, emphasizing the importance of early nutritional and clinical interventions. These findings underscore the need for comprehensive, multidisciplinary care strategies to address key prognostic factors and improve outcomes in ALS patients.

## INTRODUCTION

Amyotrophic lateral sclerosis (ALS) is a multifaceted neurodegenerative disorder marked by the deterioration of upper and lower motor neurons. It generally begins in adulthood, approximately at 55 to 75 years of age. It follows a progressive course, with a median survival of ∼ 3 years after symptom onset, though life expectancy can range from months to more than 10 years. Respiratory failure predominantly accounts for mortality. The disorder exhibits clinical and genetic heterogeneity, resulting in a broad range of pathogenic mechanisms across the spectrum of the disease. 1

To date, the disease remains incurable, with a poor prognosis. Weight loss and malnutrition emerge as significant clinical features during disease progression, resulting from reduced food intake or energy deficit due to dysphagia and/or hypermetabolism. 2 Evidence suggests that weight loss at the time of ALS diagnosis is a significant prognostic factor. Therefore, preventing or minimizing weight loss after diagnosis could extend the patient's lifespan. 3 The present study aimed to explore how demographic and clinical characteristics relate to survival in ALS patients, emphasizing the role of weight loss percentage at the time of diagnosis.

## METHODS

### Study design and population

The current is a retrospective study that used the database of the ALS Multidisciplinary Care Center at Hospital Universitário Onofre Lopes (HUOL), in the city of Natal, state of Rio Grande do Norte (RN), Brazil, covering the period from January 2008 to March 2022. Additional data and cohort characteristics have been published previously. 4 The study was approved by the Ethics in Research Committee of HUOL (CEP HUOL/UFRN 3540209).

### Data collection

Patients with ALS were referred to the center by neurologists or general practitioners. This center, an outpatient clinic within the Brazilian health network, is the only ALS treatment facility in RN. These patients were diagnosed following the revised El Escorial criteria. 5 Subsequently, each patient underwent regular follow-up appointments every 3 to 4 months, which included those with the nutrition team. The initial nutritional assessment was conducted at the time of diagnosis.

Data on sex, age at disease onset, disease progression rate according to the revised ALS functional rating scale (ALSFRS-R), 6 survival, usual weight, and current weight were extracted from the computerized database of the ALS Multidisciplinary Care Center. This database contains prospectively-collected clinical data from all ALS patients followed at the center.

Disease onset was recorded as the moment when the patient or family first noticed signs/symptoms of muscle weakness. Survival was defined as the time, in months, from the disease onset to death or tracheostomy, whichever occurred first, or until the censoring date (March 31, 2023). Progression rate was calculated as: (48 - ALSFRS-R score at the time of diagnosis)/duration from onset to diagnosis in months. The results were categorized into slow (< 0.5), intermediate (0.5–1.0), and fast (> 1.0) progression rates. 7 The percentage of weight loss was used to assess the weight change of the patients at the time of diagnosis (early weight loss). This variable was calculated as: (usual weight - current weight)/usual weight × 100. Subsequently, the patients were categorized into subgroups of percentage of weight loss ≥ 10% and < 10%.

Cases originating from locations outside of RN, those lacking usual or current weight recordings, those with incomplete documentation on disease onset or submission to gastrostomy, as well as patients with irregular follow-up were excluded from the analysis.

### Statistical analysis

Associations involving the categorical variables were determined by the Chi-squared (χ ^2^ ) test. Quantitative averages were compared using the Mann–Whitney U test. Survival was calculated from symptom onset to death/tracheostomy or censoring date; it was analyzed using the Kaplan-Meier method and compared with the log-rank test. Multivariable analysis for survival was performed with the Cox proportional hazards model. Statistical analysis was performed using the IBM SPSS Statistics for Windows (IBM Corp.) software, version 28.0. A significance level of 5% was adopted for all analyses.

## RESULTS

### Demographics, clinical characteristics, and weight loss

A total of 132 patients were included in the study; however, some variables (progression rate and submission to gastrostomy) had missing data. Associations of demographic and clinical characteristics of the participants with the percentage of weight loss at the time of diagnosis are presented in Table 1 . The mean age of the participants at symptom onset was of 56.9 ± 12.1 years, with a higher proportion of male patients, accounting for 59.8% of the cases. Most participants presented the spine as the site of onset (62.1%), had been submitted to gastrostomy (55%) and non-invasive ventilation (72.7%), and presented intermediate or fast disease progression rates (76.8%). The median time from the onset of symptoms to gastrostomy placement was of 24 (interquartile range [IQR]: 17–38) months. The minority of them were tracheostomized (34.8%) and presented weight loss ≥ 10% (40.9%) at the time of diagnosis.

**Table 1:** Association of participants' demographic and clinical characteristics with the percentage of weight loss at the time of diagnosis

Characteristics		Total ( *n* = 132)	Percentage of weight loss		*p* -value ^a^
			< 10% ( *n* = 78)	≥ 10% ( *n* = 54)	
Sex: n (%)	Male	79 (59.8)	50 (64.1)	29 (53.7)	0.231
	Female	53 (40.2)	28 (35.9)	25 (46.3)	
	Total	132 (100)	78 (100)	54 (100)	
Age at disease onset: n (%)	< 60 years	77 (58.3)	51 (65.4)	26 (48.1)	**0.048**
	≥ 60 years	55 (41.7)	27 (34.6)	28 (51.9)	
	Total	132 (100)	78 (100)	54 (100)	
Site of onset of amyotrophic lateral sclerosis: n (%)	Bulbar	50 (37.9)	21 (26.9)	29 (53.7)	**0.002**
	Spinal	82 (62.1)	57 (73.1)	25 (46.3)	
	Total	132 (100)	78 (100)	54 (100)	
Submission to gastrostomy: n (%)	No	58 (45)	35 (45.5)	23 (44.2)	0.891
	Yes	71 (55)	42 (54.5)	29 (55.8)	
	Total ^b^	129 (100)	77 (100)	52 (100)	
Submission to non-invasive ventilation: n (%)	No	36 (27.3)	22 (28.2)	14 (25.9)	0.773
	Yes	96 (72.7)	56 (71.8)	40 (74.1)	
	Total	132 (100)	78 (100)	54 (100)	
Tracheostomy: n (%)	No	86 (65.2)	50 (64.1)	36 (66.7)	0.761
	Yes	46 (34.8)	28 (35.9)	18 (33.3)	
	Total	132 (100)	78 (100)	54 (100)	
Disease progression rate: n (%)	Slow	30 (23.3)	23 (30.3)	7 (13.2)	**0.013**
	Intermediate	49 (38)	29 (38.2)	20 (37.7)	
	Fast	50 (38.8)	24 (31.6)	26 (49.1)	
	Total ^b^	129 (100)	76 (100)	53 (100)	

Notes: ^a^ Chi-squared; test; values in bold indicate statistical significance ( *p* < 0.05). ^b^ Fewer than 132 participants due to missing data.

No significant association was found between the percentage of weight loss and sex, submission to gastrostomy, submission to non-invasive ventilation, and tracheostomy. However, those with weight loss ≥ 10% at the time of diagnosis were older (≥ 60 years) ( *p* = 0.048) and presented bulbar onset ( *p* = 0.002) and faster disease progression rate ( *p* = 0.013) ( Table 1 ).

### Survival and prognostic factors

Associations of demographic and clinical characteristics of the participants with the outcome are presented in Table 2 . No significant association was found between the outcome and sex or site of ALS onset. However, a significantly higher proportion of participants who experienced the outcomes of death or tracheostomy (indicating shorter survival) were older at the disease onset (≥ 60 years) ( *p* = 0.012), had experienced weight loss ≥ 10% at the time of diagnosis ( *p* = 0.016), presented faster disease progression rate ( *p* < 0.01), and had been submitted to either gastrostomy ( *p* < 0.01) or non-invasive ventilation ( *p* = 0.010) ( Table 2 ).

**Table 2:** Association of participants' demographic and clinical characteristics with the outcome of interest

Variables		Outcome of interest: death or tracheostomy		Total	*p* -value ^a^
		Yes	No		
Sex: n (%)	Male	56 (70.9)	23 (29.1)	79 (100.0)	0.735
	Female	39 (73.6)	14 (26.4)	53 (100.0)	
Age at disease onset: n (%)	< 60 years	49 (63.6)	28 (36.4)	77 (100.0)	**0.012**
	≥ 60 years	46 (83.6)	9 (16.4)	55 (100.0)	
Site of onset of amyotrophic lateral sclerosis: n (%)	Bulbar	39 (78.0)	11 (22.0)	50 (100.0)	0.228
	Spinal	56 (68.3)	26 (31.7)	82 (100.0)	
Weight loss at diagnosis: n (%)	< 10%	50 (64.1)	28 (35.9)	78 (100.0)	**0.016**
	≥ 10%	45 (83.3)	9 (16.7)	54 (100.0)	
Disease progression rate: n (%)	Slow	13 (43.3)	17 (56.7)	30 (100.0)	**< 0.01**
	Intermediate	35 (71.4)	14 (28.6)	49 (100.0)	
	Fast	44 (88.0)	6 (12.0)	50 (100.0)	
Submission to gastrostomy: n (%)	Yes	63 (88.7)	8 (11.3)	71 (100.0)	**< 0.01**
	No	29 (50.0)	29 (50.0)	58 (100.0)	
Submission to non-invasive ventilation: n (%)	Yes	75 (78.1)	21 (21.9)	96 (100.0)	**0.010**
	No	20 (55.6)	16 (44.4)	36 (100.0)	

Notes: ^a^ Chi-squared; test; values in bold indicate statistical significance ( *p* < 0.05).

**Figure 1:**
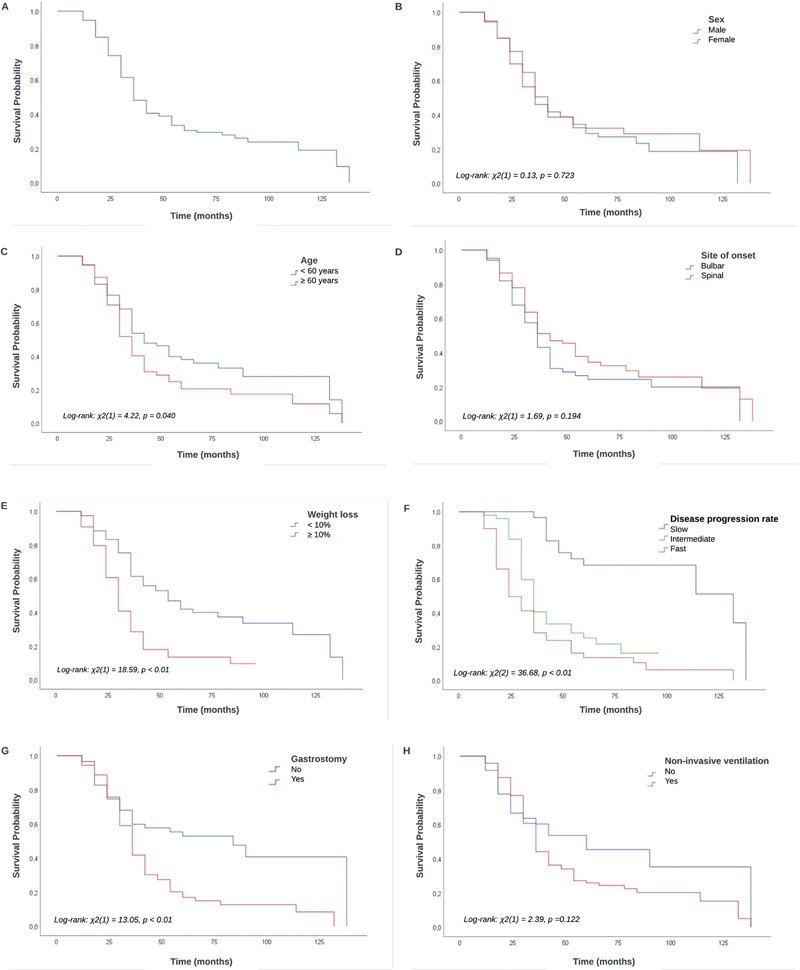
Kaplan-Meier cumulative survival ( **A** ), and survival curves by sex ( **B** ), age ( **C** ), site of onset ( **D** ), weight loss ( **E** ), disease progression rate ( **F** ), use of gastrostomy ( **G** ), and use of non-invasive ventilation ( **H** ) of patients with amyotrophic lateral sclerosis.

The survival analysis evaluated 132 cases of patients with ALS with a mean follow-up of 41.5 ± 28.4 months and a median of 32 (IQR: 22.0–57.8) months. Of the total of 132 patients, 72% presented the outcome of interest (death or tracheostomy). The participants presented an overall survival of 90.9% at 12 months, of 65.7% at 24 months, and of 44.1% at 36 months of follow-up. The median survival time was of 34 (95%CI: 30.9–37.1) months ( Figure 1A ).

Survival was shorter for individuals with age ≥ 60 years ( Figure 1C ), weight loss ≥ 10% ( Figure 1E ), intermediate and fast disease progression rates ( Figure 1F ), and those submitted to gastrostomy ( Figure 1G ). No significant difference in survival was observed regarding sex, site of onset, and submission to non-invasive ventilation ( Figures 1B , 1D , and 1H ).

In the multivariable analysis, we observed that patients aged ≥ 60 years were 1.66 times more likely to reach the outcome of interest (95%CI: 1.07–2.58). Subjects with early weight loss ≥ 10% were 2.06 times more likely to achieve the outcome of interest (95%CI: 1.32–3.21). Patients with intermediate and fast progression rates were 4.17 (95%CI: 2.04–8.55) and 5.83 times (95%CI: 2.93–11.60) more likely to reach the outcome of interest respectively. Finally, individuals submitted to gastrostomy were 1.98 times more likely to present the outcome of interest (95%CI: 1.25–3.15) ( Table 3 ).

**Table 3:** Characteristics of the patients associated with the outcome of interest (death or tracheostomy) identified by multivariate analysis

Characteristics	HR _G_^a^	95%CI	*p* ^b^	HR _A_^a^	95%CI	*p* ^b^
Age at disease onset ≥ 60 years	1.52	1.01–2.28	**0.045**	1.66	1.07–2.58	**0.024**
Weight loss at diagnosis ≥ 10%	2.42	1.59–3.69	**< 0.001**	2.06	1.32–3.21	**0.001**
Intermediate disease progression rate	3.86	1.93–7.71	**< 0.001**	4.17	2.04–8.55	**< 0.001**
Fast disease progression rate	6.31	3.23–12.33	**< 0.001**	5.83	2.93–11.60	**< 0.001**
Submission to gastrostomy	2.24	1.42–3.53	**< 0.001**	1.98	1.25–3.15	**0.004**

Abbreviations: HR _G_ , gross hazard ratio; HR _A_ , adjusted hazard ratio.

Notes: ^a^ HR _G_ and HR _A_ : hazard Ratios calculated using the Cox regression model. ^b^ Significance by the Wald Chi-square test; values in bold indicate statistical significance ( *p* < 0.05).

## DISCUSSION

The present study investigated the associations involving demographic and clinical characteristics and survival in ALS patients, focusing on the percentage of weight loss at the time of diagnosis. We found that older age, bulbar onset, and faster disease progression were associated with weight loss ≥ 10% at diagnosis. Additionally, age at disease onset, weight loss at diagnosis, faster disease progression, and submission to gastrostomy or non-invasive ventilation were associated with the outcome of interest (death or tracheostomy). Out of 132 patients, 72.% experienced the outcome of interest, and the median survival time was of 34 months (2.8 years). Survival was shorter for individuals aged ≥ 60 years, those with weight loss ≥ 10%, intermediate to fast disease progression, and those submitted to gastrostomy. Among these factors, disease progression rate and weight loss at diagnosis were the strongest predictors of shorter survival.

Our results on the negative impact of weight loss ≥ 10% at the time of diagnosis, associated with factors such as older age, bulbar onset, and faster disease progression, are supported by other studies. 8 9 This association may be attributed to age-related vulnerability, 10 loss of appetite, 11 high prevalence of dysphagia, reduced food intake, 12 13 and hypermetabolism or energy deficits, 14 particularly in ALS patients with bulbar onset. These factors collectively worsen nutritional status and prognosis.

It is well established that the nutritional status of ALS patients significantly impacts their prognosis, underscoring the importance of nutritional care as a core component of multidisciplinary treatment. 15 For the anthropometric nutritional assessment of ALS patients, guidelines recommend simple measurements such as of the body mass index (BMI) and weight loss percentage to monitor nutritional status regularly. 16 17 Since BMI does not capture body composition or quantify weight loss, 9 18 the percentage of weight loss is more predictive of prognosis. Studies 3 9 19 indicate that weight loss ≥ 10% at onset or during disease progression is a significant prognostic factor for survival in ALS patients.

The current study demonstrated survival probabilities of 90.9% at 12 months, 65.7% at 24 months, and 44.1% at 36 months, with a median survival from onset of 34 months. Millul et al. 20 reported survival rates of 78% at 12 months, 56% at 24 months, and 32% at 48 months, as well as a median survival from onset of 39.2 months. Pupillo et al. 21 observed a 1-year survival of 76.2% and a median survival of 26 months. Other studies 22 23 have reported variations in median survival rates among ALS patients. These differences may be attributed to variations in study design, patient demographics, access to care, availability of multidisciplinary treatments, and advancements in palliative care. In the present study, survival was shorter for individuals aged ≥ 60 years, those with ≥ 10% of weight loss, and those with intermediate or fast disease progression. These findings are corroborated by those of other studies. 9 18 24 25

In the current study, we found an association between submission to gastrostomy and shorter survival. Research on this topic has yielded mixed results. Some studies 9 26 27 have linked gastrostomy to prolonged survival in ALS patients, while a recent Cochrane review 28 of 23 non-randomized studies noted a lack of well-designed randomized trials to investigate this issue. This review 28 highlighted the limited availability of high-quality evidence supporting tube feeding in ALS, despite endorsements from international experts and guidelines. This inconsistency may result from several factors, including the timing of the placement of the percutaneous endoscopic gastrostomy (PEG) tube, respiratory function status, severity of dysphagia, age, nutritional status, and the adequacy of energy and nutrient intake.

A retrospective study 29 aimed to investigate a comprehensive flow of 188 ALS patients before and after PEG tube placement. The authors 29 highlight the critical timing for PEG tube insertion in ALS patients. They calculated a recommended time point for gastrostomy (T-rec), which was defined as the earlier time point between a weight loss of more than 10% and advanced dysphagia indicated by the ALSFRS-R swallowing subscore ≤ 2. Their analysis revealed a T-rec of 22 months after symptom onset; however, there was an 8-month delay between the T-rec and the actual PEG placement, which was associated with a rapid decline in the ALSFRS-R score. 29 Another study 30 did not find a survival benefit from gastrostomy, likely due to the procedure being performed too late. Delays in PEG tube placement are often linked to disease progression and worsening symptoms, including dysphagia, bronchial aspiration, weight loss, and nutritional decline. These factors profoundly affect the prognosis and should be carefully evaluated alongside the timing of PEG placement. Another factor that may affect the progression of the disease is the cognitive status, which should be evaluated and considered when making decisions regarding the timing of invasive interventions.

Additionally, discussions about gastrostomy and ventilation options can be emotionally challenging for ALS patients, requiring sensitive and empathetic communication. Decisions on gastrostomy involve patient and family preferences, support systems, socioeconomic factors, and healthcare access. The healthcare team should explain the risks and benefits clearly, prioritizing patient autonomy and informed decision-making. 31 A multidisciplinary approach with regular symptom monitoring should guide discussions on the optimal timing for PEG insertion, as the timing of the procedure can significantly impact postprocedure survival and overall patient outcomes.

Respiratory function is a critical factor influencing the risk of ventilatory complications during PEG placement, potentially affecting patient prognosis. However, individual cases can be effectively managed by an experienced multidisciplinary team. Even ALS patients with severe ventilatory impairment can safely undergo PEG placement with nasal non-invasive ventilation (NIV) support. 26 32 A risk stratification tool combined with procedural adaptations for PEG placement has been proposed to enhance safety for higher-risk ALS patients. 33 Respiratory function can independently influence the survival time of ALS patients, especially in those with delayed PEG placement.

The progression of dysphagia closely mirrors the advancement of ALS, and it is associated with reduced food intake, dehydration, and compromised nutritional status. In the study by López-Gómez et al., 34 dysphagia was observed in 78% of the patients, with 38.8% receiving a PEG. Their findings suggest that early PEG placement not only reduces hospital admissions related to dysphagia complications but also offers significant benefits for patients who might otherwise delay the procedure. Despite the variety of tools and assessments available to evaluate dysphagia, there is still a need to identify the most effective method to guide decisions on PEG insertion in ALS patients, based on the extent of swallowing deterioration. 35 These considerations prompt us to reflect that the long-term benefits of PEG may be compromised by severe dysphagia, malnutrition, and the advanced stages of ALS.

Studies have shown that age, bulbar onset, and poor nutritional status are linked to decreased survival in ALS patients. Age-related vulnerability not only accelerates disease progression 10 but may also increase the risk of complications during or after PEG placement. 36 Improved nutritional status and minimal weight loss prior to PEG placement have been associated with better survival outcomes. In a prospective cohort study, the median survival following gastrostomy was of 12 months for ALS patients who experienced 10% or less of weight loss since diagnosis, while those who lost more than 10% of their body weight presented a median survival of 7.7 months. 37 This highlights that the patient's nutritional status at the time of PEG placement plays a significant role in determining survival outcomes following the procedure. Discussing PEG insertion before significant weight loss or severe dysphagia develops is crucial, and minimizing the delay between recommendation and insertion should be a priority. Efforts should focus on reducing delays, and compromised respiratory function should not prevent patients from undergoing the procedure. 29

The adequacy of energy and nutrient intake is the final, yet equally important, factor influencing survival time after PEG placement in ALS. The adequacy of energy and nutrient consumption following gastrostomy has a significant impact on the patient's nutritional status and, consequently, survival. Theoretically, the immediate benefits of PEG are adequate food intake and weight stabilization; 26 however, in practice, many factors can influence this adequacy, such as vomiting, regurgitation, constipation, diarrhea and gastric stasis, abdominal distention, and feeding tube obstruction. 38 Achieving adequate energy and nutrient intake through homemade feeding in home enteral nutrition can be challenging. While homemade enteral feeding has certain advantages, it often provides lower caloric density, insufficient levels of nutrients, and it is related to a higher number of complications compared with the industrialized formulas. 39 However, industrialized formulas, despite being nutritionally superior, are costly, particularly in resource-limited settings or developing countries. 39 Moreover, even when using industrialized formulas, failing to meet patients' nutritional requirements can result in energy and nutrient deficits, ultimately leading to weight loss and malnutrition.

While timely PEG placement is essential to address dysphagia and nutritional deficits in ALS, the procedure alone does not guarantee adequate energy and nutrient intake. During a 12-month follow-up, 40 a high-calorie diet in ALS patients with gastrostomy showed potential for improving nutritional status and extending survival. Effective nutritional support depends on an individualized dietary plan and its proper implementation following gastrostomy. Furthermore, the progression of ALS, which varies greatly among individuals, also significantly impacts prognosis. Therefore, survival outcomes cannot be attributed solely to the performance or timing of gastrostomy without considering the critical factors previously discussed.

Most participants in the present study, who are assisted at our center, are covered by Brazil's federal public health system and come from lower socioeconomic backgrounds. This is associated with limited access to food and nutritional supplements. Most patients' families are required to go to court to obtain industrialized enteral formulas for their dietary plan after PEG placement. Given these challenges, we believe many participants in the current study were unable to meet their energy and nutrient requirements after PEG placement, which may have influenced their survival time after the procedure, alongside other potential factors.

An important aspect to consider is the use of the El Escorial criteria for patient inclusion in the present study. If we had used the more recent Gold Coast criteria, with improved diagnostic sensitivity and predictive value, we might have enabled the inclusion of a broader spectrum of ALS cases, particularly those with bulbar-predominant presentations. Given that bulbar-onset ALS is associated with a more aggressive course and higher mortality, its increased representation under the Gold Coast criteria could influence survival estimates and the observed impact of weight loss. We chose to use the El Escorial criteria because the Gold Coast criteria, while designed to enhance early diagnosis in clinical settings, may carry a higher risk of overdiagnosis, potentially including cases of ALS-mimic syndromes. Moreover, the El Escorial criteria remain the standard in clinical research, ensuring greater diagnostic specificity within study cohorts. Future studies incorporating the Gold Coast criteria may offer further insights into the relationship involving weight loss, disease progression, and survival, while also assessing the potential impact of broader diagnostic inclusion.

In conclusion, the current study highlights the significant impact of weight loss and disease progression on survival in ALS patients, emphasizing the importance of early nutritional and clinical interventions. The findings underscore the critical need for comprehensive, multidisciplinary care strategies to address key prognostic factors and improve patient outcomes. However, the study has several limitations, including its retrospective design, incomplete data on progression rate and submission to gastrostomy, and reliance on data from a single reference center in Brazil. Additionally, information on the adequacy of energy and nutrient intake was not collected. Despite these limitations, epidemiological studies examining the relationship involving demographic and clinical characteristics and survival in ALS patients in Brazil remain scarce. The present study provides valuable insights into patient outcomes, particularly the role of weight loss percentage at diagnosis and its influence on survival following PEG placement. Future multicenter cohort studies with larger sample sizes are needed to deepen our understanding of these critical issues. Our findings reinforce the importance of timely, targeted interventions and multidisciplinary care to improve the prognosis and quality of life for ALS patients.
